# Kodama-XUUB: an informative classification for alveolar echinococcosis hepatic lesions on magnetic resonance imaging

**DOI:** 10.1051/parasite/2021062

**Published:** 2021-09-27

**Authors:** Éléonore Brumpt, Wenya Liu, Tilmann Graeter, Paul Calame, Shi Rong, Yi Jiang, Weixia Li, Haihua Bao, Éric Delabrousse

**Affiliations:** 1 University Bourgogne Franche-Comté (UFC) and Besançon University Hospital, WHO Collaborating Centre on Prevention and Treatment of Human Echinococcosis/National French Reference Centre for Echinococcosis, UMR 6249 CNRS-UFC Chrono-environment 25030 Besançon France; 2 Xinjiang Medical University, First Affiliated Hospital, WHO Collaborating Centre on Prevention and Care Management of Echinococcosis 830000 Urumqi Xinjiang Uyghur Autonomous Region PR China; 3 Ulm University Hospital, Department of Diagnostic and Interventional Radiology Albert-Einstein-Allee 23 89081 Ulm Germany; 4 Qinghai University, Qinghai University First Affiliated Hospital 810001 Xining Qinghai Province PR China; 5 Department of Anatomy, University of Franche-Comté 25000 Besançon France; 6 Nanomedicine Laboratory, INSERM EA 4662, University of Franche-Comté 25000 Besançon France

**Keywords:** Alveolar echinococcosis, Magnetic resonance imaging, Kodama classification, XUUB study

## Abstract

*Objective*: To propose a modification of the Kodama classification to classify type III lesions of alveolar echinococcosis (AE) that do not have microcysts. *Materials and Methods*: 200 magnetic resonance imaging (MRI) images of AE liver lesions from four endemic regions of the world were classified according to Kodama, distinguishing within type III those with microcysts from those without. Each center included 50 MRIs of patients with unoperated AA liver lesions. The first 50 cases were classified by a first reader in the presence of four second-line readers from each region. Then each second-line reader classified his or her 50 cases. *Results*: In all centers, type III lesions were predominant: 58% of the total lesions and 23% of them were without microcysts. The average age of the patients was 47 years. In China, the patients were on average younger and the lesions larger. German patients had more lesions within the liver. Type I and II lesions, synonymous with earlier diagnosis, were more common in Europe. *Conclusion*: The Kodama classification needed to be modified because of the existence of a significant proportion of unclassifiable lesions. This is especially true since the presence of microcysts is an informative element of parasite activity. Therefore, this study proposes a Kodama-XUUB classification with type IIIa lesions having microcysts and type IIIb lesions not having microcysts.

## Abstract (fr)

*Objectif* : Proposer une modification de la classification de Kodama permettant de classer les lésions de type III qui ne présentent pas de microkystes. *Matériels et Méthodes* : 200 IRM de lésions hépatiques d’EA en provenance de quatre régions d’endémie dans le monde ont été classées selon Kodama, en distinguant au sein du type III celles qui présentaient des microkystes de celle qui n’en présentaient pas. Chaque centre a inclus 50 IRM de patients avec lésions hépatiques d’EA non opérées. Les 50 premiers cas ont été classés par un premier lecteur en présence des quatre seconds lecteurs issus de chacune des régions. Puis chaque second lecteur a classé ses 50 cas. *Résultats* : Dans tous les centres, les lésions de type III étaient largement prédominantes : 58 % du total des lésions et 23 % d’entre elles ne présentaient pas de microkystes. La moyenne d’âge des patients était de 47 ans. En Chine, les patients étaient en moyenne plus jeunes et les lésions plus larges. Les patients allemands présentaient plus de lésions au sein du foie. Les lésions de type I et II, synonymes de diagnostic plus précoce étaient plus fréquentes en Europe. *Conclusion* : La classification de Kodama nécessitait d’être modifiée du fait de l’existence d’une proportion non négligeable de lésions non classifiables. Ce d’autant que la présence de microkystes est un élément informatif de l’activité du parasite. C’est pourquoi cette étude propose une classification Kodama-XUUB avec un type IIIa de lésions avec microkystes et un type IIIb sans microkystes.

## Introduction

Alveolar echinococcosis (AE) is a tumor-like parasitic liver disease of the northern hemisphere caused by the larval stage of *Echinococcus multilocularis* [16, 29]. This infection is a major public health problem in parts of Central and Eastern Europe, China, Russia, Turkey, Central Asia, and Northern Japan [12]. In 2010, Torgerson et al. [25] estimated its incidence at 18.235 (11.900–28.200) new cases per year, including 16.629 (91%) in China, making it one of the most important diseases in some rural regions of China, such as the Tibetan plateau. Without treatment, mortality attributed to AE exceeds 90% in 10–15 years [25, 26] as opposed to cystic echinococcosis (CE), caused by *E. granulosus sensu lato*, with higher incidence but lower mortality [12, 16, 29]. Its transmission is linked to the ingestion of water or food infected by parasite eggs. Several risk factors are recognized, such as owning a cat or dog, being in contact with foxes, being a farmer or walking in the forest, especially in endemic areas [10]. The metacestode of *E. multilocularis* develops nearly exclusively in the liver as a pseudo-tumor, but may subsequently reach other organs by contiguity or by remote metastasis formation [16]. The latent phase of AE, before being symptomatic, can exceed 10 years; this is why in 1/3 of cases in Europe, the discovery of the disease is incidental following a medical work-up for fatigue, weight loss or hepatomegaly, through an ultrasound examination or a standard blood test. At this stage, the pseudo-tumor may be responsible for long lasting non-icteric or icteric cholestasis or abdominal pain or be revealed by hepatic biliary or vascular complications, or symptoms related to metastases. All such symptoms may mimic any type of liver or biliary disease [16, 20, 30]. The diagnosis of AE is based on a combination of clinical, epidemiological, and radiological evidence, and positive serology, and confirmed by histopathological examination and/or nucleic acid detection of *E. multilocularis* in the lesions [7]. The reference treatment is complete surgical resection whenever possible, associated with anti-parasitic treatment, albendazole or mebendazole, for 2 years. In all other cases, anti-parasitic treatment is indicated over the long term, in many cases for life [27].

Imaging plays a crucial role in the diagnosis and monitoring of the disease [16]; it is key for early diagnosis in asymptomatic patients and a basis for therapeutic strategy [27, 30]. Ultrasound is part of the first-line diagnostic procedures but requires an experienced operator. In most cases (70%), ultrasound reveals a lesion of pseudo-tumoral appearance, with hypoechoic and hyperechoic portions, irregular edges which are the sites of hyperechoic calcifications, and a central necrotic hypoechoic zone [3, 18]. Computed tomography (CT) characterizes the calcifications and provides essential anatomical information for surgical treatment. PET-CT identifies the intense inflammatory response around the lesion by the increased uptake of 18F-fluorodeoxyglucose, and thus reveals parasitic activity indirectly, in the absence of immune disorders which can disrupt peripheral inflammatory response to the lesion [8]. As a result, although it is responsible for significant irradiation [4], PET-CT has become the routine examination used for patient follow-up in countries where it is available [7, 8, 21]. Magnetic resonance imaging (MRI) provides more precision for the characterization of hepatic AE lesions: assessment of solid portions, cystic (or more precisely “pseudocystic”) portions due to central necrosis, microcysts that are pathognomonic of the disease, and macrocalcifications. The presence of microcysts appears to be strongly correlated with PET-CT hypermetabolic activity around AE liver lesions [2] and thus suggests that this non-radiating examination could be very useful for patient follow-up [19]. In 2003, Kodama et al. proposed to classify AE lesions from type I to type V through MRI identification of solid and/or cystic portions in the lesions [17]. However, this classification has never been systematically used and evaluated in a significant number of patients recruited in different regions of the world. We still need to determine its relationship, if any, with the natural history of the disease, with or without treatment.

Our primary objective was to evaluate the need for modification of the Kodama classification by analyzing liver MRIs of AE patients from 4 endemic regions in Europe and China. Our secondary objectives were to evaluate inter-reader agreement of the classification and to evaluate the relationship between the different types of the modified classification and the developmental stages of the metacestode/disease progression.

## Materials and methods

### Study centers

A multi-center study was conducted involving four academic and world-class reference AE medical research centers: Xining and Urumqi in China, Ulm in Germany, and Besançon in France (XUUB consortium). These centers are all located in areas where AE is endemic. Xining is the capital of Qinghai province in western central China, Urumqi, which is located further northwest in China, is the capital of the Xinjiang Uyghur autonomous region. Ulm is located in southwestern Germany in the Baden-Wurttemberg region and Besançon is a city in northeastern France, the capital of Franche-Comté.

### Kodama classification

**Figure 1:**
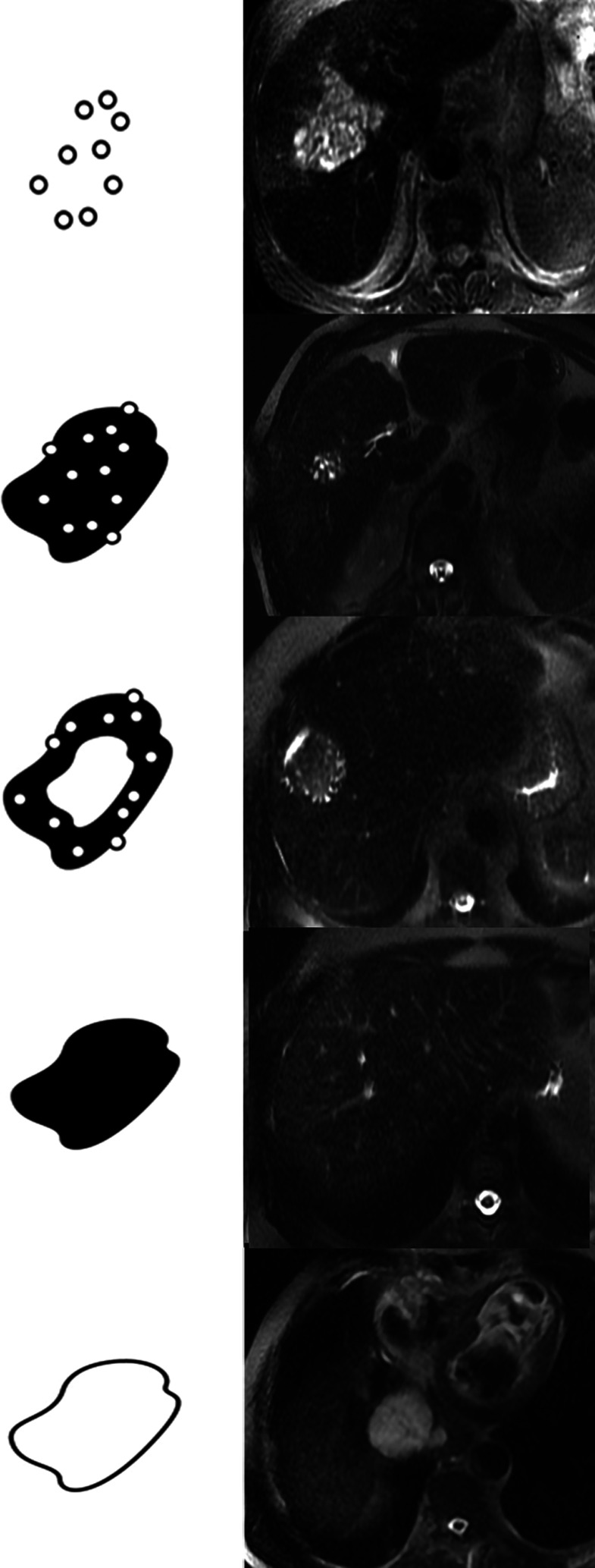
Schematic drawings (left) and T2-weighted MR images (right) of AE lesions show (top–down) the five types in the Kodama classification: type 1 multiple small round cysts without a solid component; type 2 multiple small round cysts with a solid component; type 3 a solid component surrounding large and/or irregular cysts with multiple small round cysts; type 4 a solid component without cysts; type 5 a large cyst without a solid component.

The Kodama classification was published in the journal Radiology in 2003 and is based on the MR appearance of hepatic AE lesions on T2-weighted sequences [17]. It classifies the hepatic AE lesions into 5 types according to the presence or not of a solid and/or cystic portion within the lesion (Fig. 1). Lesions with multiple small, rounded cysts (which we will call “microcysts”, according to the recommendations of the International Association of Echinococcosis) without a solid component are classified as type I. The presence of a solid component in addition to the small rounded microcysts classifies the lesion as type II. A lesion with a large and irregular pseudocystic area surrounded by a solid component and small rounded microcysts is classified as type III. The absence of a pseudocyst and the sole presence of a solid portion classify the lesion as type IV. Type V is a larger, fully pseudocystic lesion, without a solid component and without microcysts.

### Inclusion and exclusion criteria

All first 50 consecutive AE cases diagnosed from 2004 to 2015 in the 4 centers and that had hepatic AE lesions visible and analyzable by MR on T2 and T1 sequences injected at portal time were included in the study. No included patients had undergone previous liver surgery. No patient was excluded from the study. The patient lists were anonymized for each center; their ages and sexes were known and analyzed, but there was no direct identification of the cases available at the analysis stage.

### Imaging and data collected

Two hundred hepatic MRIs were analyzed: 50 from Xining, 50 from Urumqi, 50 from Ulm, and 50 from Besançon. The first reader (ED) has 20 years’ experience in reading liver MRIs of patients with AE and perfect knowledge of the Kodama classification. First, he classified the 50 cases from his own center in the presence of the 4 second-line readers of each center for the purpose of teaching and training them. The first reader then classified the lesions from the 150 MRIs from the other centers. Finally, upon returning to their home center and after a minimum period of one week between training and reading, each second-line reader classified the EA liver lesions of the 50 MRIs from his/her own center.

At the end of the process, a third reading was performed by the second-line readers in order to separate type III lesions from those that are close to type III but do not have microcysts, and which were classified as type III in the absence of a suitable type. Type III lesions were thus separated into two subtypes: subtype IIIa as initially defined by the classification (i.e. a lesion with a large and irregular pseudocystic area surrounded by a solid component and small rounded microcysts), and subtype IIIb possessing all the characteristics of type III but without microcysts.

The T2 sequence was used to classify the lesion (whenever single) or the two largest lesions (whenever there was more than 1 lesion) according to the Kodama classification. The T1 sequence injected with gadolinium made it possible to count the number of lesions, to measure the size of the largest lesion by its largest diameter, and to determine whether there was vascular and/or biliary invasion; study of the vascular or biliary involvement, which are not included in the criteria for Kodama classification, is part of a distinct study.

### Statistical analysis

The categorical data were expressed as number and percentage and compared by Pearson Chi^2^ or Fisher’s exact test. Continuous variables were expressed as mean and standard deviation and compared with Student’s test when the distribution was normal, and expressed as median and interquartile range and compared by Wilcoxon test when the distribution was not normal. Inter-reader agreement for Kodama classification between first and second-line reader was analyzed with weighted kappa statistics (0.00–0.20, slight agreement; 0.21–0.40, fair; 0.41–0.60, moderate; 0.61– 0.80, substantial; 0.81–1.00, almost perfect). A value of *p* < 0.05 was considered statistically significant with a 5% probability of error. All analyses were performed with R, version 3.4.4 (R Core Team 2017).

## Results

### Characteristics of the study population

The patients with hepatic AE included in the study were 47 years old on average (±18.63). Patients from European endemic areas were older, with the highest average age in Besançon [62 years-old (±16.43)] and the lowest in Xining [36 years old (±12.36)]. In Xining, no patients were over 60 years old and only 4 (8%) sin Urumqi. In Ulm, the over-60s represented 48% of patients, and 62% in Besançon. Fifteen patients (30%) were over 81 years old in France, only one patient in Germany (2%), and no Chinese patients were over 81 years old (Table 1).

**Table 1:** Characteristics of the study population.

	XUUB total	Xining	Urumqi	Ulm	Besançon	*p*-value	Europe	China	*p*-value
	*n* = 200	*n* = 50	*n* = 50	*n* = 50	*n* = 50		*n* = 100	*n* = 100	
Sex						0.048			0.777
Male	97 (49)	20 (40)	27 (64)	19 (38)	31 (62)		50 (50)	47 (47)	
Female	103 (51)	30 (60)	23 (46)	31 (62)	19 (38)		50 (50)	53 (53)	
Age	47.1 ± 18.6	36 ± 12.4	36.9 ± 13.1	53 ± 17.5	62.5 ± 16.4	<0.001			<0.001
< 18 years	7 (4)	4 (8)	2 (4)	1 (2)	0 (0)		1 (1)	6 (6)	
18–40 years	60 (30)	20 (40)	27 (54)	11 (22)	2 (4)		13 (13)	47 (47)	
41–60 years	74 (37)	26 (52)	17 (34)	14 (28)	17 (34)		31 (31)	43 (43)	
61–80 years	43 (22)	0 (0)	4 (8)	23 (46)	16 (32)		39 (39)	4 (4)	
> 81 years	16 (8)	0 (0)	0 (0)	1 (2)	15 (30)		16 (16)	0 (0)	
Number of lesions	1 (1–2)	1 (1–2)	1 (1–2)	1 (1–4)	1 (1–2)	0.248	1 (1–2)	1 (1–2)	0.091
1 lesion	162 (81)	46 (92)	44 (88)	32 (64)	40 (80)		81 (81)	90 (90)	
2–3 lesions	30 (15)	4 (8)	5 (10)	12 (24)	9 (18)		21 (21)	9 (9)	
4–6 lesions	6 (3)	0 (0)	1 (2)	4 (8)	1 (2)		5 (5)	1 (1)	
> 6 lesions	2 (1)	0 (0)	0 (0)	2 (4)	0 (0)		2 (2)	0 (0)	
Size of the largest lesion	95.5 ± 50.9	102.2 ± 46.4	135.3 ± 45.8	80.8 ± 50	63.7 ± 29.6	<0.001	72.26 41.76	118.72 48.81	<0.001
Type I	35.7 ± 34.5	–	76.5 ± 43.1	19.4 ± 12.3	–		19.4 ± 12.3	76.5 ± 43.1	
Type II	67.5 ± 36	83.6 ± 31.1	108 ± 58.6	55.1 ± 27.1	59.9 ± 25.7		57 ± 26.3	93.3 ± 44	
Type III	113.4 ± 48.5	116.6 ± 46.6	145.5 ± 43.1	121 ± 42	68.4 ± 28.3		92 ± 43.7	129.6 ± 46	
Type IV	55.9 ± 28.1	56.3 ± 18.7	125	68	34.8 ± 19		41.4 ± 22.2	63.9 ± 28.8	
Type V	123.4 ± 52.4	145.5 ± 3.5	160 ± 26.5	–	75.8 ± 48.9		75.8 ± 48.9	155.2 ± 21.9	

### Inter-observer reliability of the Kodama classification

The inter-observer correlation regarding Kodama classification types by an experienced and less experienced readers after training was excellent, with a kappa coefficient of 0.97 (95% confidence interval: 0.93–0.99).

### Characteristics of the lesions size and number

In the majority of cases (162 (81%) patients), only one hepatic AE lesion was found in the liver; the highest number of patients with two or more AE hepatic lesions was in Ulm (18 patients (36%) (Table 1). The number of hepatic AE lesions ranged from 1 to 4 lesions in China (1, 14) and 1 to 15 lesions in Europe (1, 7).

The mean size of the largest hepatic AE lesion measured on MRIs, all centers combined, was 95 mm [10–258]. Hepatic AE lesions were larger in China: 102 mm [20–217] and 135 mm [46–258] in Xining and Urumqi, respectively *versus* 81 [10–223] and 64 mm [16–149] in Ulm and Besançon, respectively (*p* < 0.001, Table 1). After analysis by subtype according to the Kodama classification, highly variable lesion sizes were observed. The largest lesions were type III and V lesions for all countries: 114 mm [26–258] and 123 mm [16–191], respectively.

### International distribution of Kodama types

Type III AE lesions of the Kodama classification were predominant in all centers: 115 (57%) cases among the 200 patients in our study. There was a significant difference between the number of such type III lesion among the two endemic areas: Europe (49% of the lesions) and China (66% of the lesions; *p* < 0.022). Conversely, type II AE lesions, i.e. microcysts with solid component without central irregular pseudocyst, accounted for a significant proportion of the lesions in Germany and France: 37 (37%) cases in Europe, while it was less common in China: 16 cases (16%). This difference between the two endemic areas was also significant (*p* < 0.001) (Table 2).

**Table 2:** International distribution of the Kodama types including presence of microcyst or not in type III patients.

	XUUB total	Xining	Urumqi	Ulm	Besançon		Europe	China	
Kodama classification	*n* = 200	*n* = 50	*n* = 50	*n* = 50	*n* = 50	*p*-value	*n* = 100	*n* = 100	*p*-value
Type I	7 (4)	0 (0)	2 (4)	5 (10)	0 (0)	0.019	5 (5)	2 (2)	0.444
Type II	52 (26)	9 (18)	7 (14)	22 (44)	15 (30)	0.002	37 (37)	15 (15)	< 0.001
Type III	117 (59)	31 (62)	35 (70)	22 (44)	27 (54)	0.055	49 (49)	66 (66)	0.110
*with microcyst*	88 (44)	21 (42)	23 (46)	18 (36)	26 (54)	0.333	44 (44)	44 (44)	1
*without microcyst*	27 (14)	10 (20)	12 (24)	4 (8)	1 (2)	0.004	5 (5)	22 (22)	< 0.001
Type IV	14 (7)	8 (16)	1 (2)	1 (2)	4 (8)	0.017	5 (5)	9 (18)	0.268
Type V	10 (5)	2 (4)	5 (10)	0 (0)	4 (8)	0.128	2 (4)	6 (12)	0.149

At the third reading, 88 (44%) lesions were classified as type, whereas 27 (13%), initially classified as type III, had no peripheral microcysts and thus were classified as type IIIb. After excluding cases type IIIb from type III, the type IIIa (i.e. the original type III with microcysts) still predominated among all types of lesions in China and Besançon; however, in Germany, type II became the most frequent type with 22 (44%) lesions versus 18 (36%) type IIIa.

Type I AE lesions, consisting only of microcysts, were rare, found in only 2 (4%) cases in Urumqi and 5 (10%) cases in Ulm, as well as type IV, on both continents (14 (7%) cases in total). No type V cases which consisted only of a pseudocyst were reported in Germany; such cases, were present but rare in France (4 (8%)); they were also rare in China (6 (12%), 4 (8%) cases in Urumqi and 2 (4%) cases only in Xining).

## Discussion

By comparing data from 200 AE cases from 4 AE reference centers located in endemic areas of Qinghai and Xinjiang in China, and Germany and France in Europe, the XUUB-MRI study presented here is, to the best of our knowledge, the largest series of hepatic MRI cases studied in the literature. This study stresses how different the characteristics of the affected population are in China and Europe, including the number of AE lesions in the liver, and their size and type according to the Kodama classification. Reliability of the Kodama classification is excellent to identify the originally described five types of lesions in the liver based on their morphological characteristics on a T2 weighted MRI sequence [17]. However, not all cases could be properly classified because of the existence of a type which was not initially described by Kodama et al. These lesions shared a part of Kodama type III characteristics: large and/or irregular pseudocystic area surrounded by a solid component; but no microcysts could be observed on T2-weighted images. This point was particularly important to clarify because type III was the most common AE lesion type in the world, as also described by Kodama in the seminal study in Japan in 2003. We thus suggest the identification of 2 subtypes, IIIa and IIIb, to properly classify these cases. Taking all our MRI observations into account, we also suggest new hypotheses on the natural history of AE lesions in the liver.

**Figure 2:**
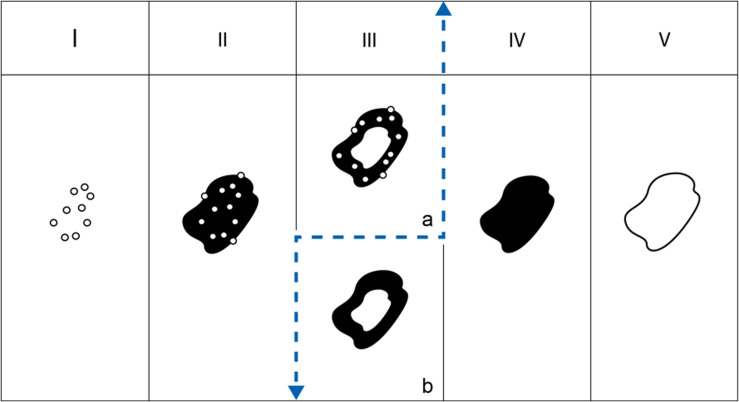
Table of the 5 types of AE lesions according to Kodama. The dashed line separates young, active lesions I, II and IIIa (left) from older, inactive lesions IIIb, IV and V (right).

Hypermetabolic activity at PET-CT is considered to be a surrogate marker of parasite viability [21] and takes a central place in patient treatment decision. A strong link between the presence of microcysts and hypermetabolic activity at PET-CT has been shown [2]. Azizi et al., however, already stressed that Kodama type III lesions without hypermetabolic activity at PET-CT had no microcysts, and conversely, all cases with microcysts exhibited hypermetabolic activity. Our study did not explore the metabolic activity of the lesions; however, it clearly showed that a number of cases, in all centers, with a central pseudocyst surrounded by a solid component had no microcysts at the periphery of the lesions; this difference may explain the original findings by Azizi et al., i.e. a greater correlation of hypermetabolic activity with the presence of microcysts, studied as such, than with the Kodama type, especially for type III for which hypermetabolic activity was observed in only 87.5% of cases. For our XUUB consortium, combined together, these observations justify the creation of two subtypes within Type III: IIIa (with microcysts) and IIIb (without microcysts) (Fig. 2) so that further studies, including PET-CT and other markers of parasite viability which are currently under study, may confirm the crucial role of the presence of microcysts in AE lesions for treatment decision. This obviously has a clinical impact that we may compare to the impact of the revision of the WHO-IWGE US classification of CE cases [14], also creating 2 subtypes of CE3: CE3A with a detached membrane, likely transitional to degeneration, and CE3B with daughter cysts in a solid matrix, active with highly viable parasite, the 2 subtypes requesting different treatment modalities [29]. In fact, for AE, the distinction between IIIa and IIIb helps to suggest that types I, II and IIIa represent “active” cases with viable parasite metacestode, shown on MRI as “microcysts”, and types IIIb, IV and V represent various stages of parasite degeneration with the disappearance of the microcysts until complete healing, without (type IV) or with (type V) a central necrotic cavity which may remain after metacestode death and be responsible for complications (especially bacterial or fungal super-infections) (Fig. 2) [15]. The slow asymptomatic development of the lesions may easily explain why the presence of a central cavity in an active lesion, thus prone to becoming symptomatic, make type III the most frequent at diagnosis in all centers, and especially in China, earlier stages (e.g. type II) being more frequently found at diagnosis in Europe where systematic imaging is routinely performed for the diagnosis or follow-up of various diseases (incidental diagnoses).

The affected population was younger in China, with larger lesions. Earlier infection of Chinese patients may explain this difference, because of the involvement of dogs in infection, while foxes are the main definitive hosts of *E. multilocularis* in Europe. Genetic differences, as well as infection when the host is still immunologically immature, may also be involved [28]. However, the larger size of lesions at diagnosis may certainly be related to the late and difficult access to care management in rural China, especially in nomadic populations of the Tibetan plateau [24] despite the ambitious government program on echinococcosis which theoretically allows free care to patients with AE. Conversely, lesions in Europe would be smaller at diagnosis thanks to easier access to screening and diagnostic tools. The larger size of type III lesions observed in the reference center of Urumqi might also be due to specific recruitment of this center, because of its longest experience in extended hepatic resections adapted to such cases, e.g. *ex vivo* liver resection with autotransplantation [1]. The combination of the large size of lesions and young age of patients may explain different strategies in the care management of the patients, and especially the frequent recourse to surgery while contra-indications to surgery are frequent among European patients because of elderly and associated conditions.

Type I was largely more frequent in Germany than in other centers. The EMUC-CT classification according to Graeter et al. highlighted the predominance of a type IV called “metastatic” in their German series of cases [13]. This type IV according to the EMUC-CT likely corresponds to Kodama type I with multiple lesions on MRI and these hepatic AE lesions with aggregation of microcysts likely represent lesions at their early stage of development, as already suggested concerning “hemangioma-like lesions” at US, frequently diagnosed on mass screening in China [3], as well as in AE recurrence after hepatectomy or transplantation [5], or in AE lesions observed in immunosuppressed patients [9]. The highest frequency of multiple and small lesions of types I and II types in Europe, and especially in Germany, where diagnosis is confirmed at an early stage, opposed to the highest frequency of single large-sized lesions of types III, IV and V types in China, and especially in Xining, where patients seek care very late, suggest that large-sized lesions with central necrotic areas may not be due to the development of a single lesion. This was anticipated in the past, but rather due to the confluence of several initial lesions likely present from the beginning of infection. There was a marked increase of cases with small multiple lesions over time in the French National Registry of AE cases, FrancEchino, established in 1982 [11]. On the other hand, the appearance of multiple foci of *E. multilocularis* metacestode is the rule after infection through the oral route [22, 23] or portal injection [31] in experimental rodents, and supports the hypothesis of a secondary confluence of small multiple lesions.

Magnetic resonance imaging limitations in the study of AE lesions mostly involve its inability to detect calcifications, which are a hallmark of AE on US and CT imaging. Recent studies have shown that several (co)schemes exist regarding calcifications and would contribute to a distinction between lesions [13]. We recently showed that the presence of specific types of microcalcifications is also, as are microcysts, highly correlated to hypermetabolic activity at PET-CT [6]. This stresses that different imaging modalities are complementary for AE diagnosis, as was repeatedly underlined in reviews on AE [16, 30] and international recommendations [7]. In our study, cases with previous surgery were excluded, but AE lesions were studied without taking the possible effect of a previous or ongoing anti-parasitic treatment into account. As far as patient’s follow-up is concerned, a specific prospective study should be designed to evaluate the possible changes in the Kodama-XUUB type of lesions during benzimidazole treatment. This type of study would help to confirm our hypotheses on the natural history of the lesions and provide better guidance for clinicians in their treatment strategies.

In conclusion, this first comparative study of hepatic AE in MRI in four AE endemic areas clearly shows that this infection affects a younger population in China than in Europe, with larger hepatic lesions. Regardless of the country, type III in the Kodama classification is by far the most common. The proposed “Kodama-XUUB” classification includes subtype IIIa with microcysts and subtype IIIb without microcysts. This refinement may allow for further studies, including PET-CT and other markers of parasite viability which are currently under study, to confirm the crucial role of the presence of microcysts in AE lesions for treatment decision-making.

## Compliance with ethical standards

The authors declare that they have no conflicts of interest.

### Human rights

The authors declare that the work described has been carried out in accordance with the Declaration of Helsinki of the World Medical Association revised in 2013 for studies involving humans.

### Informed consent and patient details

The authors declare that this report does not contain any personal information that could lead to identification of the patients.

### Funding

This work did not receive any grant from funding agencies in the public, commercial, or not-for-profit sectors.

### Author contributions

All authors attest that they meet the current International Committee of Medical Journal Editors (ICMJE) criteria for Authorship.

## Appendix

### Members of the XUUB consortium (in alphabetical order) and their affiliations

Bai Yanling (Xining, China); Bao Haihua (Xining, China); Barth Thomas FE (Ulm, Germany); Baumann Sven (Ulm, Germany); Beer Meinrad (Ulm, Germany); Beer Ambros (Ulm, Germany); Bellanger Anne-Pauline (Besançon, France); Blagosklonov Oleg (Besançon, France); Bresson-Hadni Solange (Besançon, France); Brumpt Eleonore (Besançon, France); Cao Jiayuan (Xining, China); Delabrousse Eric (Besançon, France); Demonmerot Florent (Besançon, France); Döhner Hartmut (Ulm, Germany); Fan Haining (Xining, China); Fischer Iris (Ulm, Germany); Gräter Tilmann (Ulm, Germany); Grenouillet Frederic (Besançon, France); Groß Hans-Jürgen (Ulm, Germany); Grüner Beate (Ulm, Germany); Hänle Mark M (Ulm, Germany); Henne-Bruns Doris (Ulm, Germany); Heyd Bruno (Besançon, France); Hillenbrand Andreas (Ulm, Germany); Jiang Yi (Urumqi, China); Kang Yingli (Xining, China); Kapp-Schwörer Silke (Ulm, Germany); Klein Katharina (Ulm, Germany); Knapp Jenny (Besançon, France); Koch Stephane (Besançon, France); Kratzer Wolfgang (Ulm, Germany); Li Weixia (Xining, China); Lin Renyong (Urumqi, China); Liu Wenya (Urumqi, China); Millon Laurence (Besançon, France); Montange Damien (Besançon, France); Moreau Josephine (Besançon, France); Richou Carine (Besançon, France); Schlingeloff Patrycja (Ulm, Germany); Schmidberger Julian (Ulm, Germany); Shao Yingmei (Urumqi, China); Shi Rong (Ulm, Germany); Stenger Steffen (Ulm, Germany); Theis Frauke (Ulm, Germany); Tuerganaili Aji (Urumqi, China); Turco Celia (Besançon, France); Vanlemmens Claire (Besançon, France); Vuitton Dominique A (Besançon, France); Vuitton Lucine (Besançon, France); Wang Haijiu (Xining, China); Wang Xiaoping (Xining, China); Wang Jian (Urumqi, China); Wen Shengbao (Xining, China); Wen Hao (Urumqi, China); Wu Yousen (Xining, China); Yin Guixiu (Xining, China); Zhang Wenbao (Urumqi, China).
